# Effects of visual input on changes in the bioelectrical activity of the cervical and masticatory muscles in myopic subjects

**DOI:** 10.1038/s41598-022-13607-1

**Published:** 2022-06-08

**Authors:** Grzegorz Zieliński, Anna Matysik-Woźniak, Michał Baszczowski, Maria Rapa, Michał Ginszt, Magdalena Zawadka, Jacek Szkutnik, Robert Rejdak, Piotr Gawda

**Affiliations:** 1 Department of Sports Medicine, Medical University of Lublin, Lublin, Poland; 2 Chair and Department of General and Pediatric Ophthalmology, Medical University of Lublin, Lublin, Poland; 3 Interdisciplinary Scientific Group of Sports Medicine, Department of Sports Medicine, Medical University of Lublin, Lublin, Poland; 4 Students’ Scientific Association at the Department and Clinic of General and Pediatric Ophthalmology, Medical University of Lublin, Lublin, Poland; 5 Department of Rehabilitation and Physiotherapy, Medical University of Lublin, Lublin, Poland; 6 Department of Functional Masticatory Disorders, Medical University of Lublin, Lublin, Poland

**Keywords:** Oral anatomy, Risk factors, Oral medicine, Physical examination

## Abstract

The study aimed to analyze the changes within the bioelectrical activity of the cervical spine and masticatory muscles during the change of visual stimulus—open and closed eyes test. After applying the inclusion criteria, 50 subjects were included in the study, with visual impairment ranging from −0.5 to −5.75 Diopters. Four muscle pairs were analyzed: the anterior part of the temporalis muscle (TA), the superficial part of the masseter muscle (MM), the anterior belly of the digastric muscle (DA), and the middle part of the sternocleidomastoid muscle (SCM) belly during rest, teeth clenching, teeth clenching on dental cotton rollers, and active mouth opening. Statistical analysis showed a significant decrease in the bioelectrical activity during teeth clenching of all analyzed muscles during the closed eyes test. Significant decreases of electromyographic values were also observed during resting activity within TA muscles, during teeth clenching with dental cotton rollers within SCM and DA muscles, and during active mouth opening within the right masseter. Changing the visual stimulus from open eyes to closed eyes in people with myopia affects the bioelectrical activity of the masticatory and cervical spine muscles.

## Introduction

Myopia is among the most frequently occurring diseases affecting the eyeball in the world population. Its’ prevalence is estimated from 10 to 90%, depending on the country. The largest number of people affected by myopia live in Asia^1–3^. In recent years there has been a systematic increase in the incidence of myopia. According to analyses, the number of patients with this disease will reach 4.8 billion by 2050^4^. Myopia is associated with excessive lengthening of the eyeball so that the image formed in the eyeball is projected in advance of the photoreceptors. The most common age of onset is during childhood and young adulthood^5^. According to epidemiological studies, myopia is most commonly associated with visual disability in the working population^6,7^. The most popular forms of myopia compensation are lenses and optical glasses. It is worth noting that the described refractive error is associated with complications such as cataract^8^.

Few studies have addressed the complications of myopia associated with myofascial changes. Visual disturbances lead to increased tension in the quadriceps and sternocleidomastoid muscles, which often can cause cranial tension and headaches^9–11^. Individuals with myopia compensate poor distance vision by not ergonomically positioning their head, e.g., forward extension, rotation, and tilt. This head positioning leads to increased tension in the neck muscles and shoulder girdle^9^. Head extension may be associated with thoracic outlet syndrome, in which the deep cervical flexors are weakened, and the suboccipital muscles are shortened. Long-term shortening of the suboccipital muscles can cause ischemia and complaints including headache, dizziness, tinnitus, and neck stiffness^12^.

Described above biomechanical changes in the neck and shoulder girdle muscles, may cause overloading of these segments and the formation of myofascial trigger points. This in turn will affect increased tension in the temporomandibular muscles^13^. Increased activity in the masticatory muscles may be associated with the development of temporomandibular disorders (TMDs)^14,15^.

TMDs include issues related to the masticatory muscles, temporomandibular joints, and surrounding tissues^16^. They are primarily characterized by joint and/or muscle pain, acoustic symptoms in the temporomandibular joint (TMJ), and limited mandible movements. TMDs can significantly affect the quality of life^17^, and are recognized by the World Health Organization as the third most common dental dysfunction after caries and periodontitis^18^. Patients are often unaware of their dysfunction until pain occurs, so many authors report varying percentages of people affected. The National Institute of Dental and Craniofacial Research estimates that TMDs affect between 5 and 12% of the population, more often women than men with the ratio of female to male patients with TMDs is 4.1:1^19^. The annual cost to treat TMDs patients in the United States is estimated at 4 billion dollars^20^.

In clinical observations there have been noticed changes in resting activity of the masticatory muscles caused by the change of visual stimulus. It has been reported that there is a decrease in temporalis muscle activity during closed eyes in people with myopia, in comparison to open eyes test^21,22^. However, there is lack of studies analyzing the effect of changing the visual stimulus (eyes open vs. eyes closed) during functional tasks within stomatognathic system.

The study aimed to analyze the changes within bioelectrical activity of cervical spine and masticatory muscles during the change of visual stimulus—open and closed eyes test. We hypothesize that visual input influences the bioelectrical activity of the masticatory and cervical spine muscles in myopic subjects. The alternative hypothesis has been established that visual input do not affect the bioelectric activity of the masticatory and neck muscles in people with myopia. To the best of our knowledge, this is the first study analyzing both functional and resting activity of masticatory muscles during the change of visual stimulus.

## Methods

### Participants

Seventy-nine subjects with myopia, four arch support rudiments, and complete dentition were enrolled in the study. Participants gave written informed consent to participate in this study.

Two clinical trials were conducted to qualify for the present experiment. The first was a study based on The Research Diagnostic Criteria for Temporomandibular Disorders (RDC/TMD)^16,23^. It was conducted by an experienced dentist with a specialization in dental prosthetics. During this part, the dentist evaluated the class and type of occlusion as well as the condition of the oral cavity. Subjects with TMDs based on RDC/TMD examination, class II and III according to Angle’s classification, open bite, crossbite, and oral inflammation were removed from the study. The second examination was the evaluation of ocular structures using a slit lamp. It was carried out by an experienced medical doctor specializing in eye diseases. Subjects with eye and optic nerve diseases were also removed from the study. In addition, based on the patient's medical history obtained during the subjective examination, some subjects were excluded from the study: those who reported trauma and previous surgical treatment in the head and neck region within the last 6 months of the examination, with neoplastic diseases (regardless of type and location) and pregnancy.

After applying exclusion criteria, 50 subjects were qualified for the study. This included 18 males aged 24 years ± 2 years and 32 females aged 24 years ± 3 years. According to the latest recommendations (2019), myopic individuals were defined as those with a refractive error ≤ −0.50 diopters (D)^24^. The study group included subjects with a defect of −0.5 D to −5.75 D. The mean defect value was −2 D (± 1.5 D) for the right eye and −2D (± 1.5 D) for the left eye. Moreover, the mean mandibular retraction in the study group was 47.06 mm (± 7.57 mm), lateral movements were: to the right 9.38 mm (± 1.48 mm) and to the left 9.70 mm (± 1.98 mm).

## Experiment design

The ophthalmic examination to verify the patients' defect consisted of the gold standard for measuring visual acuity in clinical practice and clinical trials that is the back-lit logarithm of the minimum angle of resolution (logMAR) which was used in the Early Treatment Diabetic Retinopathy Study (ETDRS)^25^. The results of the ETDRS array were confirmed in a Topcon KR-800 autokeratorefractometer test (Topcon, Japan). It is recognized as a rapid and accurate option for ocular screening ^26^.

Four muscle pairs were analyzed during surface electromyography (sEMG) examination:

- the front part of the temporalis muscle (TA);
- the superficial part of the masseter muscle (MM);
- the anterior belly of the digastric muscle (DA);
- the middle part of the sternocleidomastoid muscle (SCM).

The sEMG experiments were conducted between 8:00 am. and 12:00 pm. to minimize the effect of diurnal fluctuations in muscle activity. The subjects were seated in a standardized position in the dental chair before each examination; the height of the head restraint was individually adjusted to align the head, neck, and torso of the subjects. Before placing surface electrodes (Ag/AgCl with a diameter of 30 mm and a conductive surface of 16 mm—SORIMEX, Toruń, Poland), the skin was cleaned with 90% ethanol. The reference electrode was placed on the forehead where there is no typical muscle tissue under the skin^27^. The electrodes were symmetrically placed on the skin covering the examined muscles on both sides according to the course of the muscle fibers preceded by palpation of the muscles during mandibular movements. The edges of the electrodes covering the skin over a given muscle were in contact with each other to maintain constant electrode spacing. Placing electrodes was performed according to the surface EMG for non-invasive assessment of muscles (SENIAM) recommendations ^28^.

The placement of the surface electrodes was performed by the same physiotherapist with experience in electromyography measurements. The sEMG examination was carried out using a BioEMG III 8-channel electromyograph, compatible with the BioPAK measurement system (BioResearch Associates, Inc., Milwaukee, WI, USA). Before each measurement, an interference test and an sEMG Noise Test using BioPAK were performed. Electromyographic activity was recorded in the resting mandubular position and three functional activities: during maximal voluntary clenching in the intercuspal position (as hard as possible; 3 × 3 s, 2 s pause), during maximal voluntary clenching in the intercuspal position on dental rollers (as hard as possible; 3 × 3 s, 2 s pause), and during maximal mouth opening (as wide as possible; 3 × 3 s, 2 s pause)^29^. The sEMG protocol was tested by dual sEMG measurements on 10 participants. This two independent sEMG measurements were separated by 5 min of rest between activities^30^. There were no significant differences between repeated sEMG recordings in all functions analyzed (maximal clenching, maximal roller clenching, and maximal mouth opening). Recording resting activity of the masticatory muscles was carried out with eyes open and closed with a 5 min break between tests. There was a random selection of the initial test. Recording of the sEMG signal was performed without visual correction in the form of glasses and lenses. Subjects looked ahead in the open eye test^21,31^. Electromyographic signals obtained during the test were amplified as standard and cleaned of 99% linear scale noise using a digital BioPAK NoiseBuster filter. Automatic processing of the electromyographic signal based on root means square (RMS) calculation in the BioPAK program allowed us to obtain the average measurement of values, which were then used for the analysis of muscle activity.

### Statistical analysis

Data analysis was conducted using the Statistica (TIBCO Software Inc., Palo Alto, CA, USA, ver. 13.3). The distribution of data was verified using the Shapiro–Wilk test. Intra-group comparisons for normally distributed data were performed using Student’s t-test for dependent samples. When the assumption of normal distribution was not met, the Wilcoxon signed-rank test for paired samples was used. The significance level was set at *α* = 0.05. For t-test effect size was determined using the Cohen’s d method and interpreted as small (0.2), medium (0.5), and large (0.8) effect size. For the Wilcoxon Z-test effect size was calculated using correlation coefficient from 0 (no relationship) to 1 (perfect relationship)^32–34^. The results (shown in Table1) present mean values (M) and standard deviation (SD).

**Table 1:** Presenting summary of the results of bioelectrical activity (rest and clenching) of the studied muscles.

Mandibular condition	Muscle	Eyes open		Eyes closed		Statistics		
		M (µV)	SD (µV)	M	SD	Test	Test result	P value
Rest	TA-R	2.22	1.60	1.94	1.37	z	1.62	0.11
	TA-L	2.55	1.55	2.30	1.50	z	1.95	0.05* **ES = 0.28**
	TA mean	2.38	1.40	2.12	1.18	z	2.12	0.03* **ES = 0.30**
	MM-R	2.56	1.53	2.51	1.39	z	0.54	0.59
	MM-L	2.68	1.56	2.62	1.80	z	0.61	0.54
	MM- mean	2.62	1.34	2.56	1.37	z	0.43	0.67
	SCM-R	1.32	0.49	1.24	0.46	z	1.43	0.15
	SCM-L	1.42	0.49	1.39	0.51	z	1.16	0.25
	SCM-mean	1.37	0.43	1.31	0.41	z	1.17	0.24
	DA-R	1.84	0.71	1.77	0.66	z	0.55	0.58
	DA-L	1.81	0.74	1.72	0.70	z	0.48	0.63
	DA-mean	1.83	0.68	1.74	0.64	z	0.71	0.48
Clenching	TA-R	126.05	69.62	113.44	64.67	z	4.63	< 0.001*** **ES = 0.65**
	TA-L	130.17	78.15	121.82	75.46	z	3.48	< 0.001*** **ES = 0.49**
	TA-mean	128.11	71.83	117.63	67.06	z	3.95	< 0.001*** **ES = 0.55**
	MM-R	142.80	108.04	123.44	97.11	z	4.58	< 0.001*** **ES = 0.66**
	MM-L	138.90	103.25	124.57	96.74	z	3.37	< 0.001*** **ES = 0.48**
	MM-mean	140.85	103.46	124.01	94.79	z	4.25	< 0.001*** **ES = 0.61**
	SCM-R	10.79	9.47	9.04	7.84	z	4.48	< 0.001*** **ES = 0.64**
	SCM-L	10.26	8.92	8.41	6.48	z	3.94	< 0.001*** **ES = 0.56**
	SCM-mean	10.53	8.93	8.72	6.97	z	4.40	< 0.001*** **ES = 0.62**
	DA-R	20.47	15.97	17.61	12.30	z	3.17	< 0.001*** **ES = 0.47**
	DA-L	22.61	22.98	17.55	15.92	z	3.79	< 0.001*** **ES = 0.56**
	DA-mean	21.54	17.90	17.58	13.67	z	3.76	< 0.001*** **ES = 0.55**

Significant values are in bold.

*TA *the front part of the temporalis muscle, *MM *the superficial part of the masseter muscle, *SCM *the middle part of the sternocleidomastoid muscle, *DA *the anterior belly of the digastric muscle, *R *right site, *L* left site.

Significant difference: *p ≤ 0.05, **p ≤ 0.01, ***p ≤ 0.001.

The G*Power 3. 1 software was used to analyse the sample size ^35^. The following assumptions were assumed with a power value of 0.90, a value of α 0.05 and an estimated medium effect size of 0.50. Calculations showed that a sample of 44 participants would be sufficient to achieve statistical differences between matching pairs (t-test).

### Ethics approval

This study was conducted according to the Declaration of Helsinki principled. Approval was granted by the Medical University of Lublin Bio Ethics Committee (approval number KE-0254/229/2020).

### Statement

The authors attesting to informed consent from all subjects and/or their legal guardian(s) for publication of identifying information/images in an online open-access publication.

## Results

The comparison of bioelectrical activities of selected masticatory muscles and SCM showed a decrease in resting activity on all muscle groups when eyes were closed in comparison with open eyes. However, statistical analysis showed significant differences within temporalis anterior, and at TA-L (p ≤ 0.05). Statistical analysis showed a decrease in the bioelectrical activity of all analyzed muscles in the closed eye test (TA, MM, SCM, and DA) during maximal voluntary clenching in the intercuspal position. All results were statistically significant (p ≤ 0.05). All the tested muscles showed a decrease in functional activity during maximal voluntary clenching in the intercuspal position on dental rollers in the closed-eye test. However, statistical analysis revealed significant differences within SCM-R, SCM mean, DA-R, DA-L, and DA-mean (p ≤ 0.05). During maximal mouth opening in the closed eye test, a decrease in electromyographic activity was observed on all muscles. However, statistical analysis showed significant differences only within MM-R (p ≤ 0.05). The details are presented in Tables 1 and 2.

**Table 2:** Presenting summary of the results of bioelectrical activity (clenching on dental cotton rollers and maximum mouth opening) of the studied muscles.

Mandibular condition	Muscle	Eyes open		Eyes closed		Statistics	Test result	P value
		M (µV)	SD (µV)	M	SD	Test		
Clenching on dental cotton rollers	TA-R	118.11	63.86	115.61	61.40	z	0.65	0.52
	TA-L	122.15	69.28	121.23	66.02	z	0.46	0.65
	TA-mean	120.13	64.38	118.42	61.49	z	0.52	0.61
	MM-R	159.86	96.11	151.16	90.17	z	1.62	0.11
	MM-L	161.66	91.74	155.44	91.99	z	1.21	0.23
	MM-mean	160.76	90.99	153.30	88.19	z	1.46	0.15
	SCM-R	13.03	9.43	11.89	8.81	z	2.48	0.01** **ES = 0.35**
	SCM-L	12.57	7.74	11.99	7.90	z	1.83	0.07
	SCM-mean	12.80	8.32	11.94	8.16	z	2.57	0.01** **ES = 0.36**
	DA-R	23.26	14.77	20.92	12.65	z	2.04	0.04* **ES = 0.34**
	DA-L	23.60	18.24	21.53	18.73	z	2.71	0.01** **ES = 45**
	DA-mean	23.43	15.73	21.23	14.91	z	2.73	0.01** **ES = 0.45**
Maximum mouth opening	TA-R	8.39	10.15	7.36	6.56	z	1.28	0.20
	TA-L	7.36	7.37	6.89	5.99	z	0.70	0.48
	TA-mean	7.87	8.43	7.13	6.02	z	1.02	0.31
	MM-R	11.90	15.60	11.05	14.38	z	2.17	0.03* **ES = 0.32**
	MM-L	10.07	10.96	9.40	9.13	z	0.92	0.36
	MM-mean	10.98	13.12	10.22	11.47	z	1.69	0.09
	SCM-R	14.10	16.73	13.64	16.09	z	0.66	0.51
	SCM-L	14.57	21.15	14.61	22.30	z	0.20	0.84
	SCM-mean	14.33	18.67	14.13	18.98	z	0.51	0.61
	DA-R	76.38	44.23	72.62	38.97	z	1.04	0.30
	DA-L	74.58	39.05	74.30	41.36	t	0.10	0.92
	DA-mean	75.48	39.62	73.46	38.50	t	0.73	0.47

Significant values are in bold.

*TA *the front part of the temporalis muscle, *MM *the superficial part of the masseter muscle, *SCM *the middle part of the sternocleidomastoid muscle, *DA *the anterior belly of the digastric muscle, *R *right site, *L* left site.

Significant difference: *p ≤ 0.05, **p ≤ 0.01, ***p ≤ 0.001.

## Discussion

To the best of our knowledge, this is the first study analyzing the bioelectrical changes in selected masticatory and cervical spine muscles during rest, and functional activity with the change of visual stimulus—open and closed eyes test. Statistical analysis showed a decrease in bioelectrical activity on all investigated muscles in the closed eye tests. A decrease in electromyographic activity due to a change in visual stimuli has already been discussed in the literature. However, the current literature focuses only on resting activity of masticatory muscles. According to various authors, there is a noticeable decrease in activity on temporal muscles in the closed-eye test in individuals without TMDs^21,22^. An analysis performed on patients with myofascial pain and myopia, showed a decrease in bioelectrical potentials within the TA and MM during a change in visual stimulus (eyes closed)^36^. Based on the quoted studies by skeletal muscle physiology, it can be concluded that there should be a minor decrease in bioelectrical activity during teeth clenching with closed eyes^37^. It is worth noting that for a single muscle fiber, the force of contraction is proportional for the number of actin–myosin bonds formed. If the sarcomere is too short, the distance between the actin and myosin binding sites increases, and their alignment may also be distorted, which will reduce the contraction efficiency^37^. What can affect the change in muscle fiber length?—is for now the best question that can be posed. Some authors connect the muscular system of the masticatory organ and the visual organ to proprioceptive activation. Proprioception is the sense of orientation of the position of one's own organism. Receptors of mechanosensory neurons are located in joints and muscle fibers^38^. Proprioception is an essential factor responsible for modifying and maintaining proper muscle tone^39,40^. Incoming impulses from mechanosensory neurons cooperate with labyrinthine pulses to promote oculomotor muscle activity via the corticothalamic-vestibulo-ocular reflex (VOR)^21^. This connection can be summarized as follows: when there is a whole-body resting position and no visual stimuli through closed eyes, there is a decrease in muscle tone and muscle bioelectricity.

Marchili et al. also indicate neurophysiological connections in the form of a trigeminal system representing connections between somitic structures and structures derived from the gill arches, gathering proprioception from both somitic structures and oculomotor muscles. The medullary intermediate nucleus is a small perirhinal nucleus of the brainstem that integrates information from the head and neck and relays it to the solitary nucleus, where autonomic responses are generated^41^.

Another explanation for the results obtained may be a change in the tension of the facial structures. The connection between the organ of vision and the muscles of the masticatory organ takes place through the deep fascia of the orbit (Tenon's fascia) connecting with the deep fascia of the skull (cranial fascia) and through it with the temporal fascia^42^. One of the basic characteristics of fascia is its ability to adapt to mechanical stresses^43^. It is not possible to separate the role of the eye muscles from the Tenon's fascia. In the same way, any change in one element of the fascial network will affect the whole fascial structure^42^. The fascial network enables the right distribution of tension information produced by the various tissues covered or supported by the fascia, allowing the entire body system to interact in real-time^43^. This may account for the changes in bioelectrical voltages in our study observed in a fairly short time between tests. Especially in myopia, where changes in the fascial network can be observed due to elongation of the eyeball, which is often the cause of the defect in question^5,42^.

It is worth noting, that as in the case of the analysis of neurological connections, the biomechanical-fascial connection is one of the hypotheses that may explain the results obtained. Undoubtedly, there are relations between the stomatognathic system and the organ of vision. They require further studies and analyses that may allow better diagnosis and trapping of patients with diseases and dysfunctions within both systems. Dentists, ophthalmologists, and physiotherapists should be increasingly aware of this correlation for better diagnosis and treatment of patients.

The study presented here has several limitations. First, the diagnostic criteria for TMDs were changed to The Diagnostic Criteria for Temporomandibular Disorders (DC/TMDs) in 2014. However, in the presented study the previous version was used. So far there is no validated Polish version of DC/TMD, therefore RDC/TMD was used. The second limitation is the unequal number of men and women. We recommend checking this correlation on homogeneous groups. In our study, we analyzed the European white race. Due to the severity of myopia in Asian races, we recommend checking how myopia can modulate the bioelectric activity of the muscles of the masticatory organ on the representatives of this race. A final limitation was the lack of a control group of subjects without visual impairment.

## Conclusions

Changing the visual stimulus from open eyes to closed eyes in myopic subjects affects bioelectrical activity of the selected masticatory and cervical spine muscles.

## Data Availability

The datasets generated during and/or analyzed during the current study are available from the corresponding author on reasonable request.
